# Factors Associated with Metabolic Syndrome Among Middle-Aged Women in Their 50s: Based on National Health Screening Data

**DOI:** 10.3390/ijerph17093008

**Published:** 2020-04-26

**Authors:** HyungSeon Kim, YeonHee Cho

**Affiliations:** 1 Department of Nursing, Bucheon University, 56 Sosa-ro, Bucheon-si, Gyeonggi-do 14774, Korea; 2 Pharmaceutical Benefit Department, Health Insurance Review & Assessment Service, 60 Hyeoksin-ro, Wonju-si, Kangwon-do 26465, Korea; yeonhee96@hira.or.kr

**Keywords:** metabolic syndrome, middle-aged, women

## Abstract

This study aimed to identify the risk factors associated with metabolic syndrome among middle-aged women in their 50s to provide a strategy for managing the metabolic syndrome of those whose prevalence is rapidly increasing. Secondary data from the 2012 Korean National Health Insurance Service Medical check-up cohort database were analyzed. Participants included 36,582 middle-aged women in their 50s from the cohort who received a general medical check-up. The risk factors were estimated using logistic regression analysis. Metabolic syndrome was identified in 14.6% of the surveyed persons among middle-aged women in their 50s. Working women, low household income levels, country residents, high body mass index (BMI), total cholesterol of over 240 mg/dL, non-drinker, non-exerciser, history of diabetes or hypertension, and family history of diabetes were associated with increased risk of metabolic syndrome. It is necessary to prepare a strategy to increase access to health care services so that socioeconomic vulnerability does not lead to negative health behavior such as obesity and lack of physical activity. In particular, we recommend active interventions at workplaces for the working women who have a higher risk of metabolic syndrome.

## 1. Introduction

Metabolic syndrome develops mainly from insulin resistance and is defined as a cluster of metabolic abnormalities characterized by the co-occurrence of at least three of the following criteria: hypertension, high triglyceride (TG) levels, low high-density lipoprotein cholesterol (HDL-C) levels, abdominal obesity, and high fasting glucose [1]. It is known to increase the risk of chronic disease or mortality in addition to affecting the incidence of coronary artery disease [2]. In 2001, the Adult Treatment Panel III (ATP III) of the National Cholesterol Education Program (NCEP) identified metabolic syndrome as a presence of three or more of the five following components: impaired fasting glucose, high blood pressure, abdominal obesity, hypertriglyceridemia, and low high-density lipoprotein cholesterol [3].

The prevalence of metabolic syndrome among Korean adults over 20 years old has increased from 24.9% (1998) to 31.3% (2007) over 10 years [4], and was estimated to be 28.9% in 2013 [5]. In men, it increases continuously to their 50s and then decreases; in women, it increases slowly compared to men in the early stage, and then rapidly rises in the 50s [6]. This is caused by a change in hormonal secretion due to a decrease in ovarian function, which results from the menopause, a physiological aging process [7]. Considering that the average age of menopause among Korean women is about 49.7 years and the average life expectancy is about 83.8 years, more than one-third of their lives is spent in the menopause phase [8]. As life expectancy increases gradually, the postmenopausal life extends further. In addition, since health and disease status are expected to worsen as age increases, the health care of middle-aged women with high metabolic syndrome prevalence will become more important.

The causes of metabolic syndrome include high-calorie eating habits as well as loss of regular physical activity. Middle-aged women’s physical activities decrease, along with the overall degeneration of the body functions through menopause [9]. In addition, they experience psychologically and socially rapid changes in their lives due to their child’s growing independence, and show not only physical, but also psychological and emotional symptoms such as depression, anxiety, and valuelessness, which bring about a variety of health problems [10]. In particular, obese women show more stress due to low self-esteem and depressive tendency than normal-weight people. All these factors create a negative effect on eating habits, increasing the risk of eating disorders such as binging or overeating [11].

As women enter the middle aged of their life, their muscle amount decreases with age, their body fat gets stored around the intestines, such as the abdomen, and the low-density lipoprotein cholesterol increases and changes to a state that causes atherosclerosis. This condition increases the risk of metabolic syndrome along with blood glucose and insulin [7]. When this metabolic syndrome is left alone for a long time without treatment, it can bring about a close influence on the incidence of various cancers such as breast and colorectal cancers [12]. Thus, middle-aged women have to pay special attention on physical change after menopause. 

The NCEP-ATP III [3] did not aim to simply lower a person’s body weight or waist circumference; they suggested the improvement of long-term health behavioral factors, such as weight control, moderate-drinking, non-smoking, as well as the increase of physical activity and healthy eating habit, as a metabolic syndrome prevention and management strategy. It also emphasized that education and intervention programs preventing metabolic syndrome are needed before menopause [13]. It is necessary to intensively manage the risk factors of metabolic syndrome among middle-aged women in their 50s, whose prevalence increases sharply. 

In previous reports on the factors affecting the metabolic syndrome among Koreans [14,15,16,17], factors such as a family history of diabetes or hypertension, income level, education level, smoking, drinking, and physical exercise were presented. However, they have a limitation in that the research was limited to the cross-sectional survey analysis or research data covered only a limited geographic region even if prospective cohort data. In particular, it has been reported that women are more affected by socio-economic levels related to job than men [15]. However, studies focused on the working women and dependent women who have responsibility for most house work, are insufficient. In Korean society, housework is mostly taken care of by women, it is necessary to study housewives, whose main job is housework, and working women who have a double burden of housework and labor. Therefore, this study investigated the differences in characteristics between the metabolic syndrome group and the normal group of middle-aged women and the risk factors affected with metabolic syndrome based on the nationwide Health Screening data collected prospectively for housewives and working women.

## 2. Materials and Methods 

### 2.1. Study Design

This study is a secondary data analysis designed to compare the characteristics between metabolic syndrome group (MetS) and normal group (non-MetS) and to identify the factors affected with metabolic syndrome among Korean women in their 50s using the Korean National Medical check-up data by the Korean National Health Insurance Service (NHIS).

### 2.2. Setting and Sample

The NHIS Medical check-up cohort was created in 2002 to support academic studies of national health information. This includes 514,866 (10%) persons of the 5,150,000 eligible persons within the age of 40–79 who received general medical check-up in the course of 2002–2003. It contains socioeconomic variables, status of medical resource utilization, consultation and national health screening results, and status of clinic. In 2012, 483,421 of the 514,866 eligible persons contributed national medical check-up data. Among the 483,421 persons, 36,582 were middle-aged women in their 50s. The data from 36,548 persons were analyzed for logistic regression analysis, excluding 34 subjects (four for body mass index, three for total cholesterol, and 27 for drinking) who had missing values for demographic characteristics, health status, and health behavior variables.

### 2.3. Measurements

As for the study variables, general characteristics, health status, and health behavioral characteristics were selected among variables obtained from the Health Screening data by referring to items reported as factors related to metabolic syndrome in previous studies [15,18,19,20,21].

#### 2.3.1. Metabolic Syndrome Diagnosis Criteria

This study identified metabolic syndrome when three or more of the five risk factors were met according to the diagnosis criteria by the NCEP ATP III [3] and the specific values for waist circumference provided by the Korean Society for the Study of Obesity [22,23] were used to determine metabolic syndrome. The criteria for diagnosing metabolic syndrome were: (1) waist circumference: 85 cm or more around the abdomen; (2) blood pressure: systolic blood pressure of 130 mmHg or diastolic blood pressure of 85 mmHg or more; (3) blood TG level: 150 mg/dL or more; (4) fasting blood glucose level: above 100 mg/dL; and (5) high-density lipoprotein cholesterol (HDL-C): below 50 mg/dL.

#### 2.3.2. General Characteristics

In this study, the demographic characteristics included occupational status, family income, and residence. Occupational status was classified in such a way that workplace workers were identified with a ‘yes’ and the rest with a ‘no’. Income level was categorized into eleven groups based on income of subjects, which were subsequently reclassified as levels 0–6 (low), 7–9 (medium), and 10 (high). Residence was classified as ‘Seoul’ when they live in Seoul, ‘city’ when in Kwangju, Daegu, Busan, Ulsan, Incheon, Daejeon, or Sejong Metropolitan City, and ‘country’ when in the rest of the regions.

#### 2.3.3. Health Status and Health Behavioral Characteristics

We analyzed the health status and health behavioral characteristics and they included body mass index (BMI), total cholesterol in blood, low-density lipoprotein cholesterol (LDL-C) in blood, smoking status, drinking degree, exercise level, history of diabetes mellitus (DM), hypertension, stroke, and dyslipidemia, and family history of DM, hypertension, and stroke.

BMI was divided into ‘underweight’ for less than 18.5, ‘normal weight’ for less than 18.5–24.5, ‘overweight or obese’ for more than 25.0. Total cholesterol in blood was classified as ‘normal’ for less than 240mg/dL and ‘abnormal’ for more than 240 mg/dL. LDL-C in blood was classified as ‘normal’ for less than 130mg/dL and ‘abnormal’ for more than 130 mg/dL. 

Smoking status was classified as ‘smoker’ for the group who were smoking at the time of investigation, and ‘non-smoker’ for the group who never smoked or were not smoking at the time. Drinking degree was classified into ‘non-drinker’ for the group who did not drink at all, ‘high risk’ for the group who drank more than five glasses of Soju (Korean liquor with 17% alcohol) or more than three cans (330 mL) of beer at once for more than twice a week, and ‘moderate’ for the rest. Exercise level was classified to ‘walking’ for the group who had a walk for more than 30 min for more than five times in the last week, ‘more than moderate’ for the group who had a medium level physical exercise for more than 30 min for more than five times or a strenuous exercise for more than 20 min for more than three times in a week, and ‘non’ for the rest.

History of disease was classified into ‘yes’ for the group who had been diagnosed with DM, hypertension, stroke, and dyslipidemia, or treated with medication, and ‘no’ for the rest. Family history of disease was classified into ‘yes’ for the group whose family members (parents, brothers, or sisters) had been diagnosed with DM, hypertension, or stroke, or died of these diseases, and ‘no’ for the rest.

### 2.4. Ethical Considerations

This study’s research team was provided the data by the NHIS after ethical approval from the Institutional Review Board (P01-201911-21-010).

### 2.5. Statistical Analysis

The data were analyzed using SAS Enterprise Guide 4.3 (SAS Inc., Cary, NC, USA). The number of metabolic syndrome risk factors was presented as frequency and percentage. The differences in the general characteristics and metabolic risk factors between the normal group (non-MetS) and metabolic syndrome group (MetS) were identified by chi-square and independent *t*-test. Univariate logistic regression analyses were used to identify factors associated with metabolic syndrome among middle-aged women in their 50s. After that, multivariate regression analysis was performed using the variables that were found to be statistically significant the univariate logistic regression to adjust the influence between variables. The results were presented with odds ratios and 95% confidence intervals (CI).

## 3. Results

### 3.1. Number of Metabolic Syndrome Risk Factors

Table 1 shows the number of metabolic syndrome risk factors. Metabolic syndrome corresponds to three or more of the five risk factors; 5354 (14.6%) out of the 36,582 subjects were identified with metabolic syndrome. Of these, 1358 (3.7%) had four risk factors and 242 (0.7%) had five risk factors.

### 3.2. Differences in Risk Factors for Metabolic Syndrome

The waist circumference in the MetS was 83.8 cm, which was significantly higher than 75.3 cm in the non-MetS (*t* = −69.84, *p* < 0.001). The triglyceride level in the MetS was 188.3 mg/dL, which was significantly higher than 102.0 mg/dL in the non-MetS (*t* = −65.54, *p* < 0.001). High-density lipoprotein cholesterol in blood in the MetS was 47.8 mg/dL, which was significantly lower than 61.0 mg/dL in the non-MetS (*t* = 59.81, *p* < 0.001). Systolic blood pressure in the MetS was 130.9 mmHg, which was significantly higher than 118.0 mmHg in the non-MetS (*t* = −63.76, *p* < 0.001). Diastolic blood pressure in the MetS was 80.9 mmHg, which was significantly higher than 73.8 mmHg in the non-MetS (*t* = −51.27, *p* < 0.001). Fasting glucose in the MetS was 110.9 mg/dL, which was significantly higher than 93.3 mg/dL in the non-MetS (*t* = −40.37, *p* < 0.001; Table 2).

### 3.3. Differences in the Various Characteristics of the Normal and Metabolic Syndrome Groups

Table 3 shows the differences in the various characteristics of the normal and metabolic syndrome groups. Women who did not have a job (43.0%), earned low or medium income (63.5% and 26.6%, respectively), were overweight or obese (58.4%), and lived in country area (58.7%) were more likely to be the MetS than the Non-MetS. The rates of women who were ‘abnormal’ in total cholesterol (21.2%), were non or high-risk drinker (85.0% and 3.4%, respectively), and were non-exerciser (55.5%) were higher in the MetS than the non-MetS. Also, DM, hypertension, dyslipidemia, and family history of DM or hypertension were significantly associated with metabolic syndrome (p < 0.001). There was no difference in the metabolic syndrome prevalence rate according to LDL-C level, smoking, and stroke.

### 3.4. Factors Associated with Metabolic Syndrome among Middle-Aged Women in Their 50s

Table 4 shows the odds ratios (OR) and 95% confidence intervals (CI) for risk factors of metabolic syndrome. The factors influencing metabolic syndrome among middle-aged women in their 50s were job, income, residence, BMI, total cholesterol, drinking, exercise, past history of DM or hypertension, and family history of DM.

Women with a job had a 1.28 times higher (CI = 1.20–1.36) risk of metabolic syndrome than those without a job. The risk of metabolic syndrome was 0.73 times lower (CI = 0.66–0.81) in the high income group than low income group. Compared to the Seoul resident group, the risk was 1.15 times higher (CI = 1.04–1.26) in the country resident group. 

Compared to the normal weight group, the risk of the underweight group in BMI was 0.19 times lower (CI = 0.11–0.33), while that of the overweight or obese group was 4.26 times higher (CI = 3.99–4.53). The risk of metabolic syndrome in the abnormal total cholesterol group was 1.33 times higher (CI = 1.23–1.44) than the normal group. The risk of metabolic syndrome was 0.76 times lower (CI = 0.69–0.84) in the moderate drinking group than in the non-drinking group. Compared to the non-exercise group, the risk of metabolic syndrome was low in the walking exercise and more than the medium exercise group, or 0.90 times (CI = 0.82–0.98) and 0.83 times (CI = 0.77–0.90), respectively. 

In terms of history of disease, the risk of metabolic syndrome with DM was 4.13 times higher (CI = 3.67–4.66) than without DM and the risk with hypertension was 2.14 times higher (CI = 1.99–2.31) than without hypertension. Compared to no family history, a family history of DM was associated with a 1.15 times (CI = 1.04–1.27) higher risk of metabolic syndrome.

## 4. Discussion

In this study, we investigated the factors associated with metabolic syndrome among middle-aged women. The fact that women with a job had a higher risk of metabolic syndrome than those without a job was the major finding of this study. In Korea, women are responsible for most of the housework. Women who are more socioeconomically vulnerable are more likely to be unhealthy due to the burden of their work as a living as well as their role in family care including housework. In particular, it has been reported that the more vulnerable the socioeconomic level is for women, the higher the prevalence of metabolic syndrome is, unlike men [24,25]. This study also found that the group in the low-income tier had an increased the risk of metabolic syndrome compared to the group with high-income. Low-income level is likely to make people choose low-cost and high-calorie foods [26] and lack of leisure activities [25] due to limited resources. Bad lifestyles, nutritional imbalances, and stress due to these situations can lead to weight gain and increased insulin resistance, resulting in health problems such as metabolic syndrome and cardiovascular disease [27]. According to Seo et al. [15], the lower the income level of women is, the greater their risk is for all metabolic syndrome components, such as abdominal obesity, hyperglycemia, hypertension, high triglycerides, and high LDL-C.

The studies confirming the relationship between women’s occupations and metabolic syndrome indicate that the risk of metabolic syndrome is higher among female housewives than among female workers [28] or has no significant relationship [18], which is different from the result of this report. In this study, women with a job had a 1.28 times higher risk of metabolic syndrome than those without a job.

Although the causes of higher risk of metabolic syndrome among full-time housewives than in other occupational groups are not clear, it is possible that various factors such as childcare, educational stress, and nutritional imbalance are involved [19]. However, these factors are applicable to all women who have a unique social role as housewives. For women in the workplace, the risk of metabolic syndrome can be increased due to the physical and psychological stresses involved from the combination of homeworking and parenting, in addition to the work and the stress at work. In addition, one can presume that the result is due to the fact that the subjects of this study were medical check-up cohorts who had been undergoing national health screening regularly for 10 years since 2002 and they were likely to be interested in health enough to voluntarily perform health screenings unlike other housewives. According to the Korean National Health Insurance Act, both workers and dependents are eligible for national health screening examinations. In case of workers, health screening must be conducted once every two years or once a year depending on the occupation under the Occupational Safety and Health Act; however, dependents are not compulsory under the law. Therefore, the dependents of this study, who have undergone a health screening for 10 years despite not having any legal mandate, have a high awareness of the management of physical conditions and lifestyle that can cause metabolic syndrome.

Meanwhile, the results showed that the risk of metabolic syndrome was higher among rural residents than among Seoul dwellers. This inequality was also consistent with the comparison of urban and rural differences in other countries [19]. However, other studies [29,30] confirming the difference in metabolic syndrome among residential areas showed that the risk of developing metabolic syndrome among urban residents was higher than among rural residents, which is different from this study. These results seem to be based on demographic and socioeconomic factors rather than on differences in residential areas. In other words, residents of urban areas may increase their risk of metabolic syndrome due to unhealthy lifestyles related to urbanization such as decreased physical activity, eating habits, and stress [30]. On the other hand, it is estimated that the risk of metabolic syndrome in rural areas can be higher due to a relatively older age and lower socioeconomic level compared with urban areas.

Among the health status and health behavioral factors that affect the prevalence of metabolic syndrome, this study shows that some factors, such as higher BMI than normal, total cholesterol above 240 mg/dL, non-drinking, non-exercise, a history of DM, a history of hypertension, and a family history of DM, increase the risk of developing metabolic syndrome relatively.

Several studies have shown that obesity is a potent factor that increases the risk of metabolic syndrome [2,31], and this study shows support for that previous researches. Abdominal obesity increases with lower education and lower income levels [15], whereas lower socioeconomic factors among women are more closely associated with obesity [32]. Women’s obesity is especially complicated socioeconomically in that it is not easy to correct, but it is necessary to conduct metabolic syndrome prevention interventions that focus on improving the lifestyles of obese women.

Compared with the moderate drinking group, the risk of developing metabolic syndrome was increased in the non-drinking group in this study. Excessive drinking increases overall mortality, but low frequency and adequate amount intake of alcohol have a preventive effect on cardiovascular disease [33], while the relatively high prevalence of metabolic syndrome among women who do not drink alcohol has been confirmed in previous studies [16]. This is presumably because women usually do not drink an excessive amount of alcohol at once. Moderate drinking is known to contribute to lowering the risk of metabolic syndrome by increasing HDL-C levels and lowering blood glucose through improving insulin-mediated glucose uptake [34]. However, excessive drinking increases abdominal obesity [35] and blood pressure, ultimately increasing the risk of metabolic syndrome. Thus, we recommend moderate drinking to protect oneself from metabolic syndrome.

Regular physical activity and aerobic exercise are known to help prevent and treat metabolic syndrome [20]. The incidence of metabolic syndrome was decreased in the case of one having more than 180 min per week with moderate- to high-intensity [36] or higher-intensity exercise [26]. In this study, it was found that the risk of developing metabolic syndrome was reduced in the moderate- to high-exercise group who performed vigorous exercise for more than 20 min at least three times a week, or moderate-intensity exercise for more than 30 min at least five times a week. In addition, the walking group showed a lower risk compared to the non-exercise group. This finding suggests that even low-intensity physical activity among middle-aged women plays an important role in the development of metabolic syndrome, therefore it is necessary to maintain proper exercise along with improved awareness of physical activity.

Hypertension and DM are the diagnostic criteria for metabolic syndrome. For men, the disease history and family history of these two diseases have been reported as major risk factors for metabolic syndrome [16]. In this study focusing on women, it was also found that DM or hypertension, as well as a family history of DM, increases the risk of metabolic syndrome. Therefore, it is necessary to educate people on the importance of the active prevention of metabolic syndrome through healthy lifestyle management in case of DM or hypertension and the accompanying family history of disease.

Most studies emphasize social capital and social support as two major social determinants affecting health [37], particularly health of vulnerable people. Since they affect proactive identity and increase information resources, collaboration as well as collective decisions and actions are needed [38]. The more socioeconomically vulnerable women are, the lower their chances of accessing health care services are. They cannot look after their health well enough, and so they carry on an unhealthy lifestyle. All these ultimately increase the risk of developing metabolic syndrome. Additionally, working women often want to use healthcare resources but are not able to access them due to work demands. Therefore, a strategy for strengthening access to primary health care by setting a proper approach is needed, such as supporting health promotion activities at the workplace. When the priorities for the provision of primary health care services in the community are set, taking into account the complex socioeconomic situation of women, not just their income, is essential. Also, active action strategies, such as voucher offerings, are called for.

This study is meaningful in that it analyzes the data of medical check-ups and informs the factors associated with metabolic syndrome based on the results of national health screening data involving non-working women, as well as working women, and provides a guide to lower the risk of metabolic syndrome. However, this study also has some limitations. Since this study is a cross-sectional study design, it is difficult to infer causal relationship and attention is required for interpretation. Moreover, women are more socioeconomically affected than men in developing metabolic syndrome. Even though a variety of socioeconomic factors, such as education, occupation, and income, have a decisive impact on lifestyle and health, this study only reflects a few factors due to the limitations of the data. Therefore, future studies considering detailed socioeconomic factors, such as accessibility to health care services according to women’s employment status, are suggested.

## 5. Conclusions

This study investigated the risk factors associated with metabolic syndrome among middle-aged women in their 50s using National Health Screening Data by the NHIS to provide a strategy for managing the metabolic syndrome in groups where prevalence is rapidly increasing. As a result, it was found that the socioeconomic factors that increased the risk of disease among middle-aged women in their 50s were working women, low household income levels, and country residents. Further, the health status and health behavioral factors were high BMI, total cholesterol of over 240 mg/dL, being a non-drinker or non-exerciser, history of DM or hypertension, and family history of DM.

Based on the results of this study, it is necessary to prepare a strategy to increase access to health care services so that socioeconomic vulnerability does not lead to negative health behavior such as obesity and lack of physical activity. In particular, the higher risk of metabolic syndrome among working women compared to dependents suggests that consideration and active intervention at workplaces is needed, as confirmed by the results of this study.

In conclusion, the prevention of metabolic syndrome among women who are prone to neglect their health due to work and family demands and multiple-role playing is highly important. Prolonged research is imperative to form effective intervention strategies that will take into account the differences between working women and housewives.

## Figures and Tables

**Table 1:** Number of Metabolic syndrome Risk Factors.

Number of Risk Factors	*n* (%)	
0	11,765	(32.2)
1	11,865	(32.4)
2	7598	(20.8)
3	3754	(10.3)
4	1358	(3.7)
5	242	(0.7)
Total	36,582	(100.0)

**Table 2:** Comparison of metabolic syndrome risk factors between non-metabolic (Non-MetS) and metabolic (MetS) syndrome groups.

Characteristics	Non-MetS (*n* = 31,228)	MetS (*n* = 5354)	*t*	*p*
WC (cm)	75.44 ± 6.99	83.77 ± 8.22	−69.84	<0.001
Triglyceride (mg/dL)	102.00 ± 56.58	188.30 ± 93.33	−65.54	<0.001
HDL-C (mg/dL)	61.00 ± 20.46	47.84 ± 13.69	59.81	<0.001
SBP (mmHg)	118.00 ± 13.58	130.90 ± 13.77	−63.76	<0.001
DBP (mmHg)	73.83 ± 9.31	80.90 ± 9.43	−51.27	<0.001
FG (mg/dL)	93.29 ± 15.87	110.90 ± 31.18	−40.37	<0.001

MetS = metabolic syndrome group; WC = waist circumference; HDL-C = high-density lipoprotein cholesterol; SBP = systolic blood pressure; DBP = diastolic blood pressure; FG = fasting glucose.

**Table 3:** General characteristics of the subjects.

Characteristics			Total (N = 36,582)		Non-MetS (N = 31,228)		MetS (N = 5354)		χ^2^	*p*
**Demographic** **factor**	Job	No	13,112	(35.8)	10,808	(34.6)	2304	(43.0)	141.02	<0.001
		Yes	23,470	(64.2)	20,420	(65.4)	3050	(57.0)		
	Income	Low(0–6)	21,741	(59.4)	18,343	(58.8)	3398	(63.5)	110.67	<0.001
		Medium(7–9)	9525	(26.1)	8099	(25.9)	1426	(26.6)		
		High(10)	5316	(14.5)	4786	(15.3)	530	(9.9)		
	Residence	Seoul	5870	(16.0)	5145	(16.5)	725	(13.5)	54.50	<0.001
		City	10,847	(29.7)	9361	(30.0)	1486	(27.8)		
		Country	19,865	(54.3)	16,722	(53.5)	3143	(58.7)		
**Health** **status**	BMI	Underweight	843	(2.3)	829	(2.6)	14	(0.3)	3187.5	<0.001
		Normal	25,891	(70.8)	23,677	(75.8)	2214	(41.4)		
		Overweight or Obese	9844	(26.9)	6719	(21.5)	3125	(58.4)		
	Total cholesterol	Normal	30,143	(82.4)	25,923	(83.0)	4220	(78.8)	55.62	<0.001
		Abnormal	6436	(17.6)	5302	(17.0)	1134	(21.2)		
	LDL-cholesterol	Normal	21,043	(58.0)	18,027	(58.1)	3016	(57.0)	2.19	0.997
		Abnormal	15,261	(42.0)	12,989	(41.9)	2272	(43.0)		
**Health** **behavior**	Smoking	Non	36,080	(98.8)	30,816	(98.8)	5264	(98.5)	3.71	0.054
		Smoker	455	(1.2)	374	(1.2)	81	(1.5)		
	Drinking	Non	30,205	(82.6)	25,658	(82.2)	4547	(85.0)	31.85	<0.001
		Moderate	5141	(14.1)	4521	(14.5)	620	(11.6)		
		High risk	1209	(3.3)	1025	(3.3)	184	(3.4)		
	Exercise	Non	18,824	(51.5)	15,853	(50.8)	2971	(55.5)	49.14	<0.001
		Walking	6577	(18.0)	5629	(18.0)	948	(17.7)		
		More than moderate	11,181	(30.5)	9746	(31.2)	1435	(26.8)		
**Past** **history**	DM	No	35,039	(95.8)	30,403	(97.4)	4636	(86.6)	1311.89	<0.001
		Yes	1543	(4.2)	825	(2.6)	718	(13.4)		
	Hypertension	No	30,261	(82.7)	26,736	(85.6)	3525	(65.8)	1250.65	<0.001
		Yes	6321	(17.3)	4492	(14.4)	1829	(34.2)		
	Stroke	No	36,436	(99.6)	31,109	(99.6)	5327	(99.5)	1.75	0.186
		Yes	146	(0.4)	119	(0.4)	27	(0.5)		
	Dyslipidemia	No	34,854	(95.3)	29,877	(95.7)	4977	(93.0)	74.87	<0.001
		Yes	1728	(4.7)	1351	(4.3)	377	(7.0)		
**Family** **history**	DM	No	32,606	(89.1)	27,986	(89.6)	4620	(86.3)	52.24	<0.001
		Yes	3976	(10.9)	3242	(10.4)	734	(13.7)		
	Hypertension	No	30,718	(84.0)	26,396	(84.5)	4322	(80.7)	49.08	<0.001
		Yes	5864	(16.0)	4832	(15.5)	41,032	(19.3)		
	Stroke	No	33,282	(91.0)	28,413	(91.0)	4869	(90.9)	0.01	0.917
		Yes	3300	(9.0)	2815	(9.0)	485	(9.1)		

BMI = body mass index; LDL-cholesterol = low-density lipoprotein cholesterol; DM = diabetes mellitus. Note: missing values (BMI: four; total cholesterol: three; drinking: 27).

**Table 4:** Factors associated with metabolic syndrome.

Characteristics			OR (95% CI)		*p*
Intercept					<0.001
Demographic factor	Job (ref: No)	Yes	1.28	(1.20–1.36)	<0.001
	Income (ref: Low)	Medium	0.95	(0.89–1.03)	0.213
		High	0.73	(0.66–0.81)	<0.001
	Residence (ref: Seoul)	City	1.07	(0.96–1.18)	0.174
		Country	1.15	(1.04–1.26)	0.005
Health status	BMI (ref: Normal)	Underweight	0.19 (0.11–0.33)		<0.001
		Overweight or Obese	4.26	(3.99–4.53)	<0.001
	Total cholesterol (ref: Normal)	Abnormal	1.33	(1.23–1.44)	<0.001
Health behavior	Drinking (ref: No)	Moderate	0.76	(0.69–0.84)	<0.001
		High risk	0.86	(0.72–1.02)	0.094
	Exercise (ref: No)	Walking	0.90	(0.82–0.98)	0.013
		More than moderate	0.83	(0.77–0.90)	<0.001
Past history	Stroke (ref: No)	Yes	0.92	(0.58–1.46)	0.723
	DM (ref: No)	Yes	4.14	(3.67–4.66)	<0.001
	Dyslipidemia (ref: No)	Yes	1.08	(0.95–1.23)	0.253
	Hypertension (ref: No)	Yes	2.14	(1.99–2.31)	<0.001
Family history	Stroke (ref: No)	Yes	0.96	(0.86–1.08)	0.523
	DM (ref: No)	Yes	1.15	(1.04–1.27)	0.006
	Hypertension (ref: No)	Yes	1.02	(0.93–1.11)	0.694

BMI = body mass index; DM = diabetes mellitus; OR = odds ration; CI = confidence interval.
